# Responses of *Bunias orientalis* to Short-term Fungal Infection and Insect Herbivory are Independent of Nutrient Supply

**DOI:** 10.1007/s10886-022-01392-0

**Published:** 2022-11-19

**Authors:** Blaise Binama, Miriam Behrendt, Caroline Müller

**Affiliations:** Department of Chemical Ecology, Bielefeld University, Universitätsstr. 25, 33615 Bielefeld, Germany

**Keywords:** *Alternaria brassicae*. *Bunias orientalis.* Coordinated resource hypothesis. *Mamestra brassicae*. Nitrogen fertilization. Resource allocation

## Abstract

Plants have to allocate their resources in both growth and defense under different environmental challenges. Several plant species have become invasive particularly in disturbed fertile habitats, which may influence their resource allocation. We studied the effects of nitrate fertilization (low versus high) on various plant responses towards a pathogenic fungus, *Alternaria brassicae*, and a herbivorous insect species, *Mamestra brassicae*, in a population of *Bunias orientalis*, which is invasive in parts of central Europe. Aboveground biomass and leaf trichome density were enhanced in plants under high fertilization. In contrast, the short-term fungal infection and herbivory had no effect on aboveground biomass. Leaf water, nitrogen content and glucosinolate concentrations were neither affected by fertilization nor in response to antagonist attack. The total soluble sugar content, especially fructose, as well as leaf peroxidase activity increased significantly in leaves upon fungal infection, but independent of fertilization. Larval biomass gain and herbivore survival were likewise unaffected by fertilization. Our findings highlight that under conditions of high fertilization, *B. orientalis* plants allocate more resources into growth and morphological defenses than chemical defenses. In contrast, induced responses to short-term antagonist attack seem independent of nitrate availability in this population.

**Supplementary Information:**

The online version contains supplementary material available at 10.1007/s10886-022-01392-0.

## Introduction

Plants are exposed to a complex environment and must respond to both abiotic and biotic challenges. Abiotic challenges, such as different nutrient availability, have a huge impact on allocation of resources into plant growth and defense (Monson et al. 2022; Sun et al. 2020). Furthermore, biotic factors such as pathogens or herbivores lead to tissue damage (Kessler and Baldwin 2002), which has a significant impact on the plant’s physiology (Bostock et al. 2001). To cope with these challenges, a set of molecular mechanisms are activated in plants (Rejeb et al. 2014). Some of these responses are independent of the type of the inducing factor, while others are specifically induced only by certain types of antagonists (Bostock et al. 2001; Glazebrook and Roby 2018). Moreover, plant responses to biotic challenges may be differently modulated in dependence of the nutrient availability (Gorden and Adler 2013), enabling plants to invest more or less in different types of defenses (Rejeb et al. 2014). Soil fertilization can thus have a negative or positive impact on the host plant but also on different types of antagonists. However, we still do not fully understand how nutrient availability influences plant interactions with pathogenic fungi versus herbivores, particularly in species that established in new habitats with various levels of soil quality.

Nitrate and other nitrogen (N)-containing compounds are required by plants, influencing their growth and productivity (Ding et al. 2021; Ohyama 2010) as well as their defense (Lou and Baldwin 2004; Simon et al. 2010). Various hypotheses have been postulated on how different levels of nutrients affect growth versus defense (Bryant et al. 1983; Herms and Mattson 1992). For example, according to the carbon–nutrient balance hypothesis, only under sufficient nutrient supply plants may be able to invest more in defensive metabolites (Bryant et al. 1983; Cipollini et al. 2002). However, various of these hypotheses have been questioned (Hamilton et al. 2001). Instead, recently the coordinated resource hypothesis has been framed, which postulates that the presence of accumulated resources provides a safety margin for responses to future challenges, thereby balancing the trade-off between growth and defense (Monson et al. 2022). This safety margin should depend on fertilization and resources.

Nitrogen is also an important element in amino acids, which are needed for the biosynthesis of numerous specialized metabolites such as glucosinolates, a class of compounds found in Brassicales plants, that act defensive against many fungi and herbivores (Agerbirk and Olsen 2012; Halkier and Gershenzon 2006). The composition and concentration of glucosinolates differ in plants in dependence of several environmental challenges. For example, N fertilization has been found to lead to either increased or decreased concentrations or no changes in aliphatic or indolic glucosinolates, depending on the species (Chen et al. 2006; Kutyniok and Müller 2013; Li et al. 2007). Such shifts in defense levels may affect pathogen establishment and/or herbivore feeding and growth (Söchting and Verreet 2004). For example, high N fertilization increased disease severity caused by the fungus *Botrytis cinerea* in lettuce (Lecompte et al. 2013), but decreased susceptibility in tomato (Vega et al. 2015). Nitrogen fertilization can also affect peroxidase activity of leaves (Dietrich et al. 2004), which is involved in forming a boundary towards pathogen infection through polymerization (Cooper and Varner 1984). Herbivore performance and development also change in different directions due to N fertilization, differing among plant species (Fischer and Fiedler 2000; Kurze et al. 2018; Lu et al. 2007). Furthermore, N application can enhance the density of leaf trichomes (Bilkova et al. 2016), acting as a mechanical defense against herbivores (Wei et al. 2013). Moreover, plant defense responses induced by pathogens and herbivores are modulated in dependence of nutrient availability (Chen et al. 2010; Dietrich et al. 2004; Mur et al. 2017). Thus, the interplay between abiotic and biotic challenges needs to be considered when studying plant-antagonist interactions.

Fertilization and disturbance have frequently been found to act as primary drivers facilitating the establishment of invasive species in new areas (He et al. 2011; Leishman and Thomson 2005). One example is *Bunias orientalis* L. (Brassicaceae), which is invasive in parts of central Europe (Birnbaum 2006) and often found in highly fertilized areas, which may support its establishment (Steinlein et al. 1996; Tewes and Müller 2018). Populations from different origins grown in a common garden with either low or high nitrate fertilization showed an increased biomass (i.e. more and longer leaves) under high fertilization (Tewes and Müller 2018). In contrast, glucosinolate concentrations were not affected. There was also no significant effect on pathogen and herbivore load, but in individual populations, such as one from Würzburg (Germany), the pathogen load and leaf damage seemed lower in plants under high fertilization (Tewes and Müller 2018).

In the present experiment, we thus chose the population from Würzburg to study the effects of N fertilization (low versus high) on plant responses and their interactions with antagonists, a necrotrophic fungus and an herbivorous insect species, under controlled conditions. A high N availability was predicted to lead to an increase in total aboveground biomass, leaf trichome density and peroxidase activity, while constitutive glucosinolate concentrations were expected to be unaffected by fertilization in this plant species. Moreover, in response to fertilization, we expected shifts in the carbon-to-nitrogen (C/N) ratio, which determines plant quality for antagonists (Rostás et al. 2002). The fungus was expected to establish better on high-fertilized plants, leading thus to a higher leaf water loss as indicator for virulence (Tewes and Müller 2020). Moreover, plant sugars are nutritional sources for fungus growth (Solomon et al. 2003) and fungi can reduce photosynthesis, thus altering the sugar content in infected and uninfected leaf parts (Rosa et al. 2009). The fungal infection was thus expected to reduce total sugar and sucrose content and to induce peroxidase levels. The larval biomass and survival of the herbivore were expected to be lower in highly fertilized plants. Finally, high fertilization was expected to lead to a more pronounced changes in different groups of defenses.

## Methods and Materials

### Plant, Pathogen and Herbivore Rearing

Three separate sets of experiments were performed, one plant-pathogen experiment and two plant-herbivore experiments (one to test plant responses and one to measure herbivore performance), all using low or high fertilization conditions. Pathogen performance was only measured indirectly by comparing the water content of fungus-infected leaves to that of leaves treated with agar only as control. As necrotrophic pathogen, *Alternaria brassicae* (Berk.) Sacc. (Pleosporaceae) was used, which destroys the plant cells directly after infection mediated by a mycotoxin (Pedras et al. 2003) and uses the dead plant as a food source (Cho 2015). It can be virulent on different species of the Brassicaceae, including *Bunias orientalis* (Farr and Rossman 2022; Tewes and Müller 2020). As a herbivore, we used *Mamestra brassicae* L. (Lepidoptera: Noctuidae), which is a generalist herbivore previously reported to survive well when feeding on *B. orientalis* plants of invasive populations (Harvey and Gols 2011; Tewes et al. 2018). Seeds of *B. orientalis* were collected from four mother plants in Würzburg (49°50.95’N, 9°51.94’E ), where this species is considered invasive, and plants were grown from these seeds for one generation in a common garden in Bielefeld, ensuring cross-fertilization only among plants of this population (Tewes et al. 2018). Seed of the F1 were sterilized in 2% NaOCl with 0.03% Triton X-100 (Sigma Aldrich, Steinheim, Germany) and allowed to germinate in the dark. For the plant-pathogen experiment, seedlings were planted in 0.6 L pots filled with a 1:1 mixture of seedling soil (Archut Fruhstorfer Erde Typ LAT-Terra Standard Pikiererde; Hawita, Vechta, Germany) and low-nutrient, coarsely structured potting soil (C 710 with Cocopor, Stender, Schermbeck, Germany), which had been sterilized by steaming. As only few plants germinated in the first batch, a second batch of plants was grown a few days later. For the plant-insect experiment, seeds were initially placed in small pots (50 mL) filled with the seedling soil and the seedlings then were transferred to 0.6 L pots filled with the potting soil.

For each experiment, all plants were arranged randomly in a controlled growth chamber [for plant-pathogen experiment: initial three weeks in lab at room temperature, then at 22 °C, 16:8 h light:dark cycle, 75% r.h. in Perceival, CLF Plant Climatics, Wertingen, Germany) under Philips lamps (25 W; Philips GmbH, Hamburg, Germany); for plant-herbivore experiments: 24 °C; 16:8 h, light:dark cycle; 70% r.h.) under Osram Fluora lamps (L 36 W/77; Osram, Munich, Germany) in a climate chamber] and were watered three times per week. Half of the plants per experiment was assigned to one of two fertilization treatment groups (low and high). All plants were fertilized for two weeks with 10 mL and then with 15 mL of a mineral nutrient solution modified after Hoagland (Hoagland and Arnon 1950) per week. The fertilizer solution consisted of 12 nutrient salts (for details see Supplementary Material Table S1), with differences in the calcium nitrate tetrahydrate (Ca (NO_3_)_2_·4H_2_O; SIGMA, Darmstadt, Germany) concentration, with 1 mM Ca (NO_3_)_2_ for the low and 4 mM Ca (NO_3_)_2_ for the high fertilization treatment. Solutions were freshly prepared from stock solutions before each fertilization event.

The *A. brassicae* strain (CBS 102.24) was purchased from Westerdijk Fungal Biodiversity Institute (Utrecht, The Netherlands) and cultivated on low strength potato dextrose growth medium (30 g L^− 1^, Carl Roth, Karlsruhe, Germany) with 10 µg L^1^ ZnSO_4_ × 7H_2_O and 5 µg L^1^ CuSO_4_ × 5H_2_O in 9 cm Petri dishes. To stimulate conidia growth, Petri dishes with fungi were exposed to a 1:1 mixture of white light and black light (8:16 h light:dark cycle) at 20 °C.

Larvae of *M. brassicae* were taken from a colony established at Bielefeld University for several generations and reared in a climate chamber (24 °C, 16:8 h, light:dark cycle, 60% r.h.; Binder GmbH, Tuttlingen, Germany) on *Brassica rapa* var. *pekinensis* leaves.

### Plant-Pathogen Experiment

Half of the plants of each fertilization treatment were assigned to the agar control, the other half to the fungus infection group (n = 15 per treatment and group; one replicate less for control plants of the low fertilization treatment). For the inoculation of *B. orientalis* with the fungus *A. brassicae*, a method described by Tewes and Müller (2020) was adapted. The youngest fully developed leaf of each plant assigned to the fungus infection treatment was inoculated at its tip with two agar disks (7 mm diameter) with fungal material, taken from the edge of fungus culture plates (15–18 days old), containing growing mycelium and conidia. To ensure proper infection, the leaf section on which agar discs were applied was artificially pre-damaged with a needle-device and 20 µL of growth medium [26.5 g L-1 potato glucose broth (Carl Roth, Karlsruhe, Germany) with 0.03% [w/v] Triton X- 100 (stock solution: 0.32% [w/v] in millipore water, sterile), solution 1:10] were applied to the damaged area. The control plants received the same treatment, but the agar disks contained no mycelium. Four days later, the whole infected and respective control leaves were harvested and the leaf material was weighed (0.1 mg; CPA224S, Sartorius, Göttingen, Germany). For measurements of peroxidase activity, two leaf discs (diameter: 8 mm) were taken from the central area of the leaves (~ 2 cm distance from the infection sites). The remaining leaf part, including the infection sites, was used for later analyses of contents of water, C and N, glucosinolates and sugars. Leaf material was frozen in liquid nitrogen, and leaf discs stored at -20 °C, the remaining leaf parts at -80 °C. All leaf material was freeze-dried. The remaining aboveground biomass was harvested, dried at 40 °C for three days and weighed to determine the shoot dry mass.

Trichome density was only measured in response to fertilization. Therefore, two leaf disks were taken before pathogen infection from the leaf base of the youngest leaf of each plant with a cork borer (6 mm diameter) and stored at -20 °C. The trichomes were counted on the upper and lower leaf surface of both disks with a microscope (Olympus SZX16, Tokyo, Japan), and numbers from both disks were summed (separately for lower and upper leaf surface).

### Plant-Herbivore Experiment 1: Plant Induction

Half of the plants of each fertilization treatment were assigned to a control, the other half to the herbivore treatment group (n = 9 per treatment and group). For the induction experiment, all plants received two clip cages (15 mm diameter, 17 mm high, Fig. S1a) at the leaf base of each leaf of the youngest fully developed pair of leaves (in total four clip cages per plant). On plants of the herbivore group, one 15–17 d old *M. brassica* larva was placed in each of the four clip cages, while clip cages of the control group were left empty. If a larva did not eat the available leaf area or only a little, it was replaced by another larva within a period of 10 h in order to ensure the most uniform possible induction. After 48 h, 14 leaf discs of each leaf holding clip cages were cut with a cork borer (7 mm diameter) from the tip and middle in comparable position, the fresh weight determined, and material frozen in liquid nitrogen and stored at -80 ° C for later analyses of glucosinolates as well as C/N. The remaining aboveground plant material was harvested, weighed, dried in an oven (40 °C) for three days and weighed again to determine the shoot dry biomass.

### Determination of C and N Contents

Leaf samples were homogenized in a ball mill for 30 s at 30 Hz (MM301, Retsch, Haan, Germany). About 2 (± 0.1) mg was taken for analysis of the C and N contents using a C/N-analyzer (Vario MICRO cube, Elementar, Hanau, Germany).

### Determination of Glucosinolates

For glucosinolate analysis, about 10 (± 0.2) mg of leaf powder were extracted threefold in 1 ml of 80% methanol (≥ 98% MeOH; JT Baker, Munich, Germany) and 20 µl of allyl glucosinolate (Phytoplan, Heidelberg, Germany) was added as internal standard at the first extraction. Supernatants were applied to anion exchange columns [Sephadex A25 (AppliChem, Darmstadt, Germany) in 0.5 M acetic acid buffer, pH = 5.0], and columns rinsed with water and subsequently with 0.02 M acetic acid buffer (pH 5). For conversion of glucosinolates to desulfoglucosinolates, a purified sulfatase solution (sulfatase from *Helix pomatia* type H-1; 10,000 U, Sigma, Darmstadt, Germany) was added and the samples incubated for 24 h at room temperature. The columns were rinsed with Millipore water, samples dried and re-eluted in water. For the plant-pathogen experiment, desulfoglucosinolates were analyzed using a high performance liquid chromatograph (HPLC, Dionex Ultimate 3000, Thermo Fisher Scientific, Waltham, MA, USA) equipped with a C18 column (250 × 4.6 mm, 5 μm, Supelco, Bellefonte, PA, USA), coupled with a diode-array detector at 25 °C oven temperature. A gradient of eluent A (20% Millipore water) and eluent B (80% methanol) at a flow rate of 0.35 mL min^− 1^ was used. For the plant-herbivore experiment, desulfoglucosinolates were measured on another HPLC (1200 series; Agilent Technologies, Waldbronn, Germany), equipped with a shorter C18 column (150 × 3 mm, 3 μm; Supelco) at a flow rate of 1 mL min^− 1^. Glucosinolates were identified by comparing their retention times and spectra to an in-house database. Peaks were integrated at 229 nm and quantified using the response factors 1 for aliphatic, 0.5 for benzenic, and 0.26 for indolic glucosinolates, and glucosinolate concentrations calculated using the internal standard and sample fresh weight. Concentrations of different glucosinolates were summed up in total and within each group.

### Analysis of Sugars

The sugar analysis (plant-pathogen experiment) was performed according to a method modified after Kutyniok and Müller (2013). Freeze-dried leaf tissue [10 (± 0.2) mg] was extracted threefold in 1 mL buffer [2.5:1:1 (v: v: v) methanol: H_2_O: chloroform, with 0.2 mg/mL ribitol (99%, Sigma-Aldrich, Steinheim, Switzerland) as internal standard]. After centrifugation, subsamples of the supernatants were dried under nitrogen and derivatized using methoximation with O-methylhydroxylamine hydrochloride (Thermo Fisher Scientific, Karlsruhe, Germany) in pyridine (99.9%, Sigma-Aldrich, 20 mg ml^-1^) for 30 min at 50 °C, followed by silylation with *N*-methyl-*N*-trimethylsilyl-trifluoroacetamide (Macherey-Nagel, Düren, Germany) for 30 min at 50 °C. The samples were diluted in pyridine and then analyzed using a gas chromatograph coupled to a mass spectrometer (GC-MS 2010 Plus QP2020, Shimadzu, Kyoto, Japan) equipped with a VF-5 ms column (38 m × 0.25 mm x 0.25 μm, Agilent Technologies, Santa Clara, CA, USA). Samples were injected at 225 °C with a split ratio of 1:10 at a helium flow rate of 1.46 ml min^-1^. The oven temperature was set at 50 °C for 5 min, followed by a ramp (5 °C min^-1^) to 280 °C. The temperature of the transfer line was 250 °C. Samples were measured at 70 eV in electron impact ionization mode. A mixture of *n*-alkanes (C8-C40, Sigma-Aldrich) was measured under the same conditions. Analytes were identified by comparing Kováts retention indices (Kováts 1958), as well as mass spectra to reference compounds measured under the same parameters and the NIST database (NIST14, National Institute of Standards and Technology, Gaithersburg, Maryland, USA). The total ion count was used to determine the peak areas for the analytes. Peak areas of analytes belonging to the same metabolite were summed up (for fructose and glucose). Peak areas were divided by the area of the internal standard and the dry mass of the leaf sample.

### Measurement of Peroxidase Activity

To determine the peroxidase activity of leaves (plant-pathogen experiment), about 15–30 mg of frozen leaf material was homogenized and extracted in 250 µl of 100 mM Na-acetate buffer at pH 6.0 (5.77 g C_2_H_3_NaO_2_, AppliChem GmbH; 1.78 g C_2_H_4_O_2_, VWR, Prolabo, France) on ice, centrifuged, and the supernatant used for the photometric determination of the peroxidase activity, following a protocol by Rosa et al. (2020). Leaf extracts were mixed with 100 mM Na-acetate buffer in a 96 well plate (Microplate, 96 well, Thermo Scientific, Nuncleon™ Delta Surface, Denmark). A master reaction mix containing 12.5 mM H_2_O_2_ (30%, VWR), 0.306 mM guaiacol (substrate, > 98% 2-methoxyphenol, Thermo Fisher) and 100 mM Na-acetate buffer was added to all plant extracts. A dilution series of H_2_O_2_ in 100 mM Na-acetate buffer was measured in parallel. Immediately before the start of the measurements, a standard reaction mix containing 12 mM guaiacol, 1.4 mg peroxidase (horse radish peroxidase 124 U mg^− 1^, Sigma-Aldrich, Steinheim; Switzerland) and 100 mM Na-acetate buffer was added to each well of the dilution series. Plates were measured in a multi-plate photometer (Power wave 200 on COM1, Bio-Tek, USA) at 470 nm wave length for 4 min and data analyzed with KC4 software (Kinetical for Windows, Version 2.7 Rev 8).

### Plant-Herbivore Experiment 2: Herbivore Performance

The relative growth rate and survival of the herbivore was investigated using plants of the low and high fertilization treatment (n = 12 per treatment). One clip cage was placed at the tip of each leaf of the youngest, fully grown pair of leaves (two cages per plant), and provided with one larva of *M. brassicae* (second larval stage; 2–4 d old) per cage. Before the experiment began, all larvae were weighed ( ≤ ± 1 µg; ME36S, Sartorius, Göttingen, Germany). If a larva died during the first 24 h, it was replaced. The status (alive or dead) of each larva was recorded on a daily basis. If the available leaf area was almost entirely consumed, the clip cage was relocated on the same leaf to ensure that there was always enough food. After 7 d, the weight of each surviving larva was measured again, and the larvae were moved from the clip cages into gauze bags, allowing access to the entire leaf. The weight of each larva was measured once again after another seven (day 14), and a total of 19 d of observation. If a leaf was totally consumed before that period, the larva was moved to a comparable leaf on the same plant. If no comparable leaf was available, the larva was transferred to the leaf of a plant of the same fertilizer treatment on which another caterpillar had already died. The larval biomass gain of *M. brassicae* was calculated for each observation time point as the difference between the biomass at that time point and their initial biomass.

### Statistical Analyses

All statistical analyses and figures were performed with RStudio under R 4.1.2 (R Core Team 2020). For continuous data (Gaussian distribution), linear mixed models (LMMs) with the lmer function and lme4 R-package (Bates et al. 2015) were used for all traits measured in the plant-pathogen experiment. To meet the requirements of a normal residual distribution, the data were log or square root transformed, where necessary. For all traits of the plant-pathogen experiment, fertilization treatment, infection treatment and their interaction were considered as fixed factors, whereas plant batch was considered as random factor. The number of leaf trichomes (Poisson distribution), was analyzed by a generalized linear mixed model (GLMMs) using the lme4 package (function *glmer*; Bates et al. 2015).This model was then adjusted by glmm Template Model Builder (glmmTMB) with a negative binomial 1 family (Brooks et al. 2017) and the DHARMa package was used to check the model (Hartig 2020). To test the plant treatment effects on the density of trichomes, fertilization treatment, leaf side and their interaction were considered as fixed factors and batch and plant identity as random factors. The LMMs were fitted with a maximum likelihood approach, and *P* values were obtained using a type III Anova with Chi-square tests. The correlation between the fructose content (leaf including the infection site) and peroxidase enzyme activity (infected leaf without infected area) was analyzed by Spearman rank correlations in infected plants, separately for low and high fertilization.

The Shapiro-Wilk test and the Levene test (package: *car*; Fox and Weisberg 2019) were used to test for data distribution and homoscedasticity in the plant-herbivore experiment 1. If the data was normally distributed and with homogeneous variance, linear models (LM) were used, except for data on the total glucosinolate concentration, for which a generalized linear model (GLM) was used. Within the LM and GLM, fertilization treatment, herbivore treatment and their interaction were considered as fixed factors. The *P* values for all LMs were evaluated using *F* tests. The larval biomass gains of *M. brassicae* were evaluated by an LMM with fertilization treatment, time of observation and their interaction as fixed factors and plant identity as a random factor. The survival probability of *M. brassicae* was estimated using a non-parametric event time analysis according to Kaplan-Meier (package *survival*; Therneau 2022). The log-rank test was used to test if there was a significant difference in survival probability between larvae raised on plants of the two fertilization treatments.

## Results

### Effects of Fertilization and Pathogen Treatment on Plant Traits

**Fig. 1:**
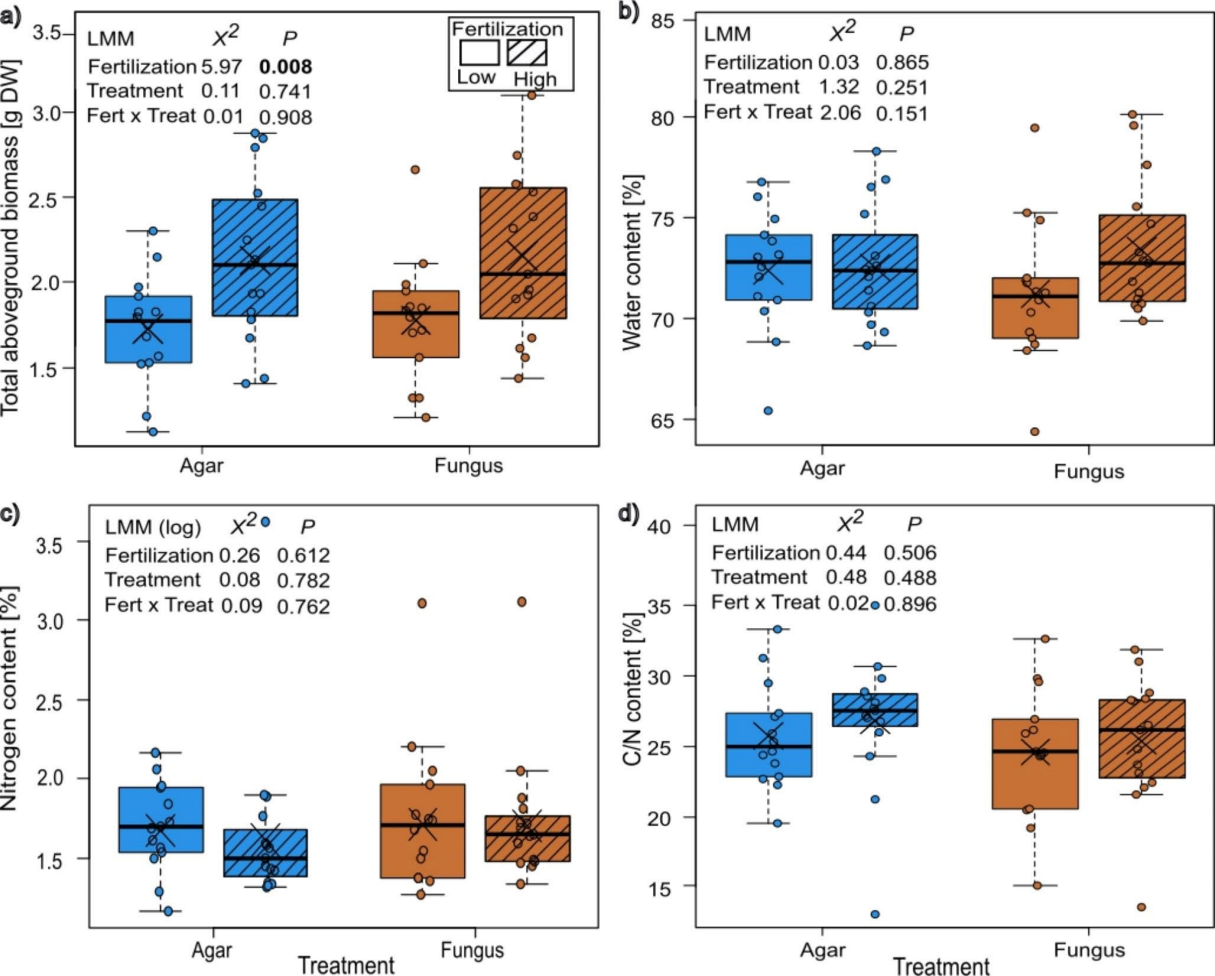
Responses of plants of *Bunias orientalis* to nitrate fertilization (Fert, low and high) and infection treatment (Treat) by *Alternaria brassicae* (control or fungus) in (a) aboveground biomass, (b) water content, (c) nitrogen content, and (d) carbon to nitrogen ratio (b-d: leaf including infection site). Data are presented as box whisker plots, boxes represent the 25th and 75th percentile and median, crosses show means, whiskers mark minimum and maximum within 1.5-fold interquartile ranges, and open dots are outliers, n = 14–15. Significant *p* values (*p* < 0.05) are highlighted in bold

In the pathogen-plant experiment, the aboveground biomass was significantly higher by about 23% in plants with high fertilization, but unaffected by fungus infection or the interaction of fertilization and infection treatment (Fig. 1a). The water content of the leaves was neither affected by fertilization, infection treatment nor their interaction (Fig. 1b). Likewise, the nitrogen content and C/N ratio were neither affected by the fertilization treatment, infection treatment, nor their interaction (Fig. 1c,d).

**Fig. 2:**
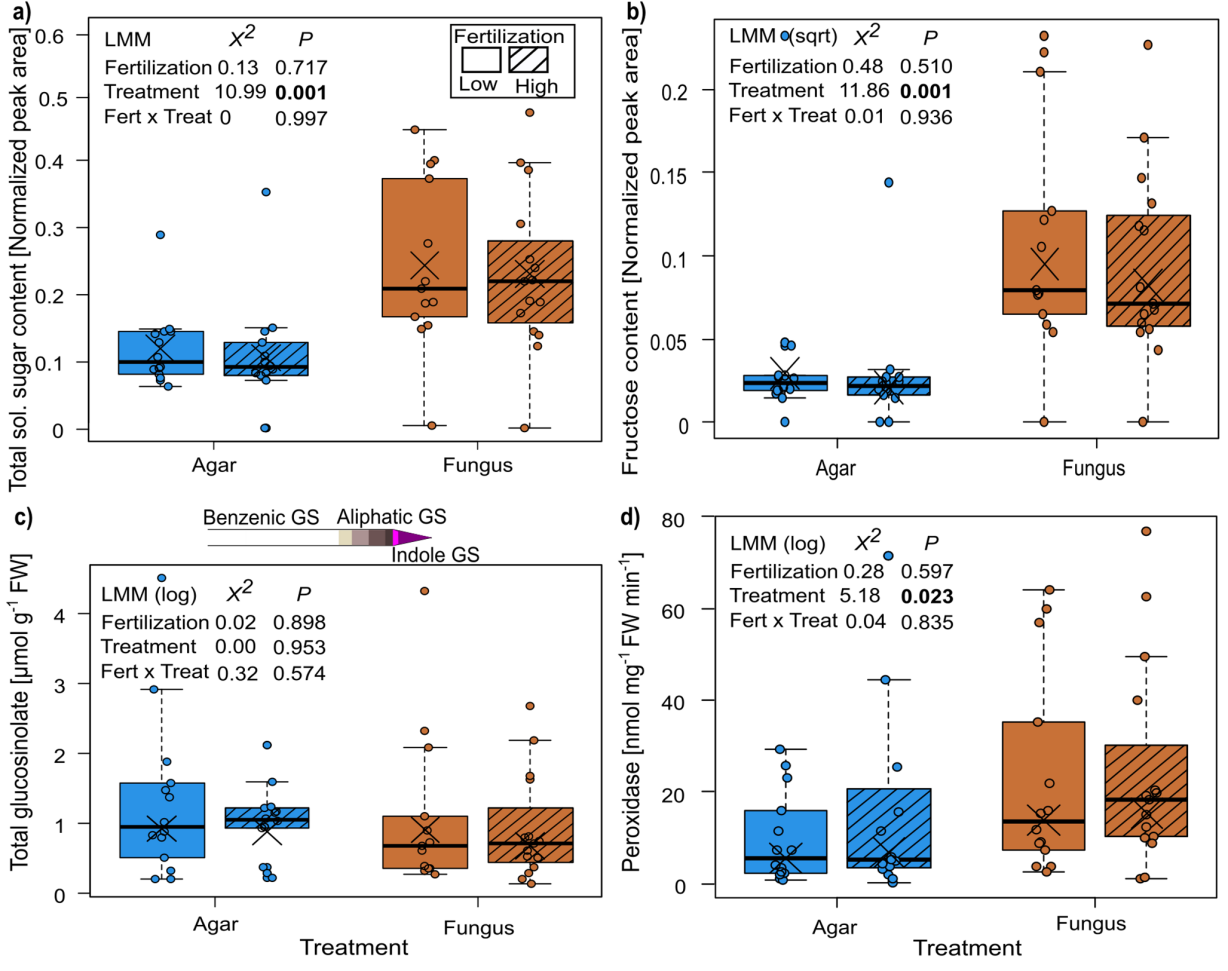
Responses of plants of *Bunias orientalis* to nitrate fertilization (Fert, low and high) and infection treatment (Treat) of leaves by *Alternaria brassicae* (control or fungus) in (a) total soluble sugar content, (b) fructose content, (c) total glucosinolate concentration [upper bar represents the identified glucosinolates, comprising one benzenic (white bar unit), four aliphatic (grey bar units) and two indole glucosinolates (purple bar units)] (a-c: leaf including infection site), and (d) peroxidase activity of control or infected leaf (d: area distant to leaf infection). Data are presented as box whisker plots, boxes represent the 25th and 75th percentile and median, crosses show means, whiskers mark minimum and maximum within 1.5-fold interquartile ranges, and open dots are outliers, one outlier (9.56) of infected leaf in low fertilization is not shown in graph (a), n = 14–15. Significant *p* values (*p* < 0.05) are highlighted in bold

The most abundant sugar in all treatments was glucose, followed by fructose, which together contributed to about 90% of all measured soluble sugars. The total soluble sugar content (normalized peak area) of the treated leaves was significantly affected by the infection treatment, being about two times higher in leaves of infected compared to those of control plants, while fertilization and the interaction of fertilization and infection treatment had no significant influence (Fig. 2a). Each of the three measured sugars exhibited a different pattern (Table S2). Similar as the total sugar content, the content of fructose was significantly affected by the infection treatment, being three times higher in fungus-infected than in control leaves (Fig. 2b). In contrast, glucose and sucrose contents were not affected by any of the treatments (Table S2).

The total glucosinolate concentration as well as concentrations of the different glucosinolate groups of the treated leaves were not influenced by any of the treatments (Table 1; Fig. 2c). The identified glucosinolates comprised one benzenic, four aliphatic, and two indole glucosinolates, with *p*-hydroxybenzyl glucosinolate being dominant (Fig. S2). Indole-3-ylmethyl glucosinolate was only present in minute concentrations and could not be detected in leaves of control plants under high fertilization. Furthermore, 4-methylsulfinylbutyl glucosinolate was only detectable in trace amounts in control plants under low fertilization (Fig. S2).

**Table 1:** Effects of fertilization and pathogen infection by *Alternaria brassicae* on leaf glucosinolate concentrations of *Bunias orientalis*

	Fertilization			Infection			Fertilization * Infection		
	*df*	χ2	*P*	*df*	χ2	*P*	*df*	χ2	*P*
Total GS (log)	1	0.02	0.898	1	0.00	0.953	1	0.32	0.574
Benzenic GS (log)	1	0.00	0.948	1	0.01	0.909	1	0.22	0.641
Aliphatic GS (log)	1	0.06	0.807	1	0.35	0.555	1	0.29	0.592
Indole GS	1	0.19	0.664	1	0.93	0.336	1	0.43	0.513

Traits were analyzed using linear mixed-effects models and values transformed as indicated, where necessary. All models were fitted using the maximum likelihood method, and *p* values were determined using likelihood ratio tests (chi-square tests). Fertilization (low or high) and Infection (control or fungus), n = 14–15, GS: glucosinolate (µmol g^− 1^ fw)

The activity of peroxidase was significantly enhanced by 36% in leaves infected by the fungus compared to control leaves, but fertilization and the interaction between fertilization and infection treatment had no significant effects (Fig. 2d). The peroxidase activity (measured in leaf area distant to the infection site) showed by trend a moderate positive correlation with the fructose content (measured in leaf including infection site), at least in plants of the low fertilization treatment (S = 166, rho = 0.544, *P* = 0.058) but not in the high fertilization group (S = 318, rho = 0.432, *P* = 0.109; Fig. S3b).

The trichome density was significantly affected by both fertilization (*χ*^*2*^ = 5.44, *df =* 1, *P* = 0.019) and leaf side (*χ*^*2*^ = 12.53, *df =* 1, *P <* 0.001), with more trichomes per mm^2^ under high fertilization and less trichomes on the upper than on the lower side (Fig. S4).

### Effects of Fertilization and Herbivore Treatments on Plant Traits

**Fig. 3:**
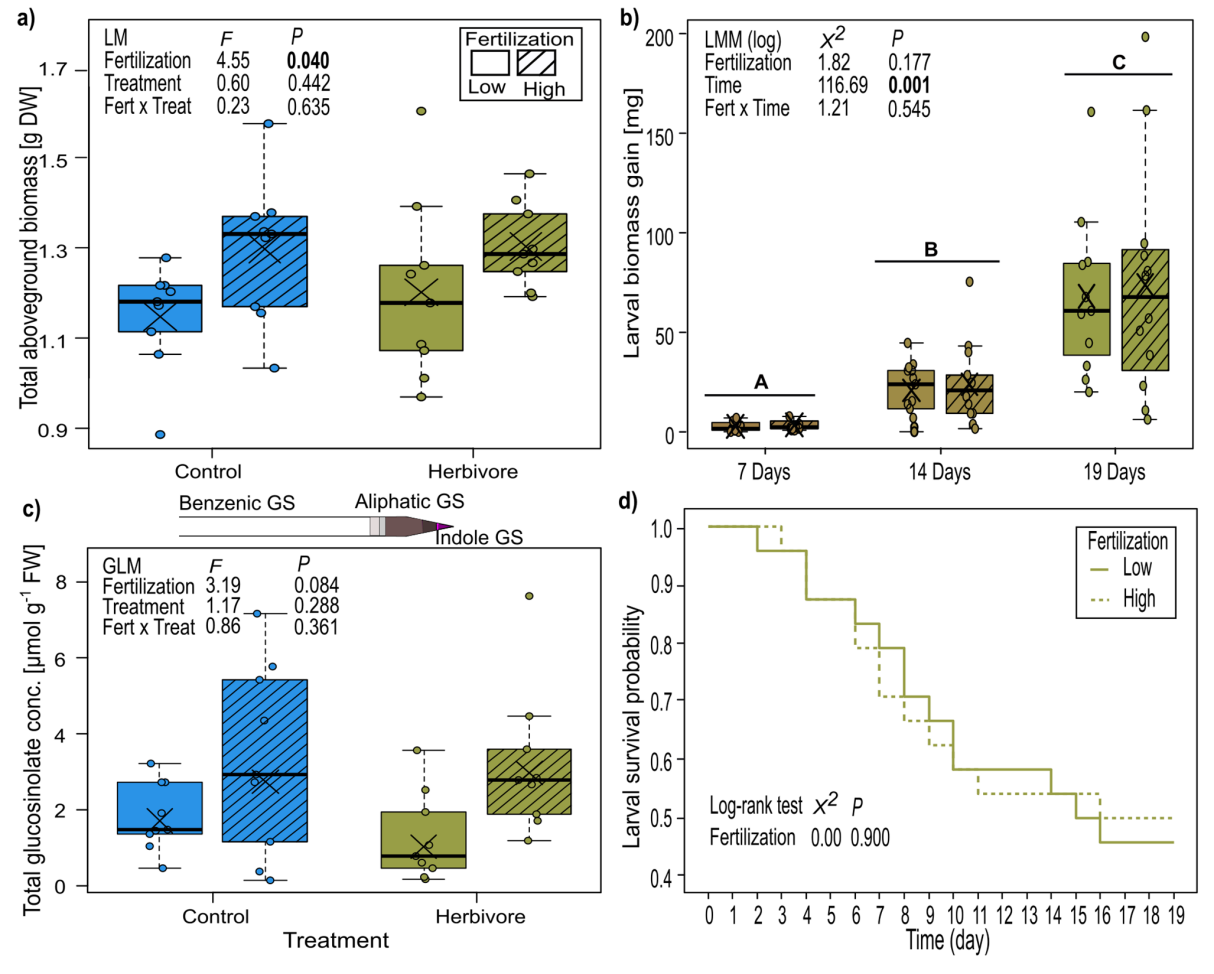
Responses of plants of *Bunias orientalis* to nitrate fertilization (Fert, low and high) and infestation treatment (Treat) of leaves by 2nd instar *Mamestra brassicae* (control or herbivore) in (a) aboveground biomass, (b) larval biomass gain (biomass at respective day minus initial biomass), (c) leaf total glucosinolate concentration [upper bar represents the identified glucosinolates comprising one benzenic (white bar unit), four aliphatic (grey bar units), and two indole glucosinolates (purple bar units)], and (d) survival probability of 2nd instar of *Mamestra brassicae* larvae reared on plants of *Bunias orientalis* grown under different nitrate fertilization (low and high). Time: day 0: n = 24 (per treatment); day 19: n = 13 for low and n = 10 for high fertilization. Survival probability was analyzed by log rank Kaplan-Meier survival analysis. Data are presented as box whisker plots, boxes represent the 25th and 75th percentile and median, crosses show means, whiskers mark minimum and maximum within 1.5-fold interquartile ranges, and open dots are outliers, n = 9. Letters above the boxes in (b) indicate significant differences among time points according to Tukey posthoc tests (*p* < 0.05). Significant *p* values (*p* < 0.05) are highlighted in bold

In the herbivore-plant experiment 1, the aboveground biomass was significantly influenced by the fertilization treatment, with plants showing on average a 10.6% higher biomass when grown under high compared to low fertilization. In contrast, herbivore infestation did not affect the aboveground biomass (Fig. 3a). Fertilization treatment had also no effect on the N content or the C/N ratio (Fig. S5a-b).

The total glucosinolate concentration as well as the concentrations of the different glucosinolate groups of the treated leaves were likewise not influenced by any of the treatments (Table 2; Fig. 3c). 4-Methoxyindol-3-ylmethyl glucosinolate was only present in minute concentrations and could not be detected in leaves of infested plants under high fertilization. Furthermore, 1-methylpropyl glucosinolate was only detectable in trace amounts in control plants under low fertilization (Fig. S6). This glucosinolate has been previously mis-identified as *n*-butyl glucosinolate, but its identity could now been confirmed with the help of a standard (kindly provided by N. Agerbirk).

**Table 2:** Effects of fertilization and herbivore infestation by *Mamestra brassicae* larvae on leaf glucosinolate concentrations of *Bunias orientalis*

	Fertilization			Infestation			Fertilization * Infestation		
	*df*	χ2	*P*	*df*	χ2	*P*	*df*	χ2	*P*
Total GS (log)	1	1.67	0.205	1	1.17	0.288	1	0.91	0.348
Benzenic GS (log)	1	1.52	0.226	1	1.60	0.215	1	1.17	0.287
Aliphatic GS (log)	1	1.64	0.807	1	0.01	0.923	1	0.03	0.867
Indole GS	1	0.82	0.372	1	2.14	0.153	1	0.06	0.805

Traits were analyzed using linear models and values transformed as indicated, where necessary. Fertilization (low or high) and Infestation Treatment (control or herbivore), n = 9, GS: glucosinolate (µmol g^− 1^ fw)

### Effects of Host Plant Fertilization on Herbivore Performance

At all three observation time points, the larval biomass gain was not influenced by the fertilization treatment of their host plants, but biomass gain significantly increased over time, with four and eleven times higher biomass gains in the second and third week of the experiment, respectively (Fig. 3b).

The survival over time was not affected by fertilization (log rank test *χ*^*2*^ = 0.01, *P* = 0.9), but dropped over time in both treatment groups (Fig. 3d).

## Discussion

This study demonstrates that aboveground biomass and morphological defense traits, i.e. trichomes, of *B. orientalis* were significantly enhanced by high fertilization. Short-term fungal infection led to several changes in phytochemical contents of the host plants but independently of the fertilization, whereas herbivore infestation did not influence any of the traits tested in this study.

Plant growth and biomass production are generally influenced by a combination of soil nutrients and various other environmental conditions (Morgan and Connolly 2013). As expected, the application of higher nitrate fertilization in this study resulted in higher aboveground biomass accumulation compared to less fertilized plants, in accordance with numerous previous studies (Anjum et al. 2012; Guo et al. 2019; Hsu et al. 2009). However, aboveground biomass was not impacted by the infection of the pathogenic fungus *A. brassicae*, which may be due to the fact that the infection treatment only lasted for four days. An enhanced nitrate fertilization is considered to increase plant susceptibility to necrotrophic fungi (Long et al. 2000), which could reduce the nutrient availability for plant growth (Berger et al. 2007; van Dijk et al. 2021). Such impacts on plant growth may only become visible after longer growth periods with the fungus. Moreover, fungal pathogens frequently lead to a reduction in the leaf water content by damaging the leaf surface (Grimmer et al. 2012) and thus evoking wilting. In this study, infected leaves did not differ in their water content compared to leaves from uninfected plants. In other populations of *B. orientalis*, a higher water loss in leaves infected by *A. brassicae* was found in plants of a native population (Rize, Turkey) compared to an invasive population (from Jena, Germany) after a 8 d infection period (Tewes and Müller 2020). Differences in the chemical and morphological defenses between different populations of *B. orientalis* but also in the infection time and specific infection conditions may affect the plant susceptibility towards this fungus. Furthermore, plant metabolism of the *A. brassicae* mycotoxin may play a role in susceptibility. The synthesis of the N-containing mycotoxin likely depends on N availability (Wang et al. 2009). Thus, modulation in virulence caused by plant fertilization may significantly influence plant susceptibility.

Against our expectation, leaf N content and C/N ratio of *B. orientalis* leaves were not affected by the fertilization treatment. This stability may indicate a balance of physiological processes regardless of nutrient availability in this plant population, at least at the used fertilizer concentrations. In contrast, in various other species, an increase in N content and decline in C/N ratio have been reported following N addition (Esmeijer-Liu et al. 2009; Lü et al. 2012; Novotny et al. 2007). Both N and C/N ratio were also not affected by the fungus infection in the present experiment. The resources accumulated during growth may have provided a safety margin also under low fertilization conditions, buffering a strong response to the fungal infection challenge, as proposed in the coordinated resource allocation hypothesis (Monson et al. 2022).

While no changes were visible in C/N, the content of total soluble sugars and in particular of fructose was significantly enhanced in *B. orientalis* leaves infected by *A.brassicae*, in contrast to our expectation. Pathogen infection is usually expected to reduce the content of sugars in plant tissue due to negative impacts on photosynthesis (Morkunas and Ratajczak 2014). Moreover, necrotrophic pathogens act as sink for the sugars and cause an increased demand of assimilates for the plant to induce defense responses (Berger et al. 2007; Liu et al. 2022). However, the impact of fungi on plant sugar contents varies significantly across plant-pathogen interactions and contents in infected leaves were found to be lower, higher or unaffected, depending on the system (Berger et al. 2007; Bonfig et al. 2006; Scharte et al. 2005). Higher levels of sugars due to infection, as also found in tobacco plants infected with the potato virus Y (Herbers et al. 2000), may be explained by physical disturbance of the transport system or stimulation of cell wall defense responses.

The leaf glucosinolate concentrations of *B. orientalis* were unaffected by fertilization. This finding is in line with previous results, as a similar N-fertilization also did not affect glucosinolate concentrations in *B. orientalis* plants of different populations grown in a field experiment (Tewes and Müller 2018). However, the concentration of glucosinolates was also not affected by the fungus treatment in our study. In contrast, in *Arabidopsis thaliana*, the concentrations of certain glucosinolates and their hydrolysis products peaked one day after leaf infection by the necrotrophic fungus *Sclerotinia sclerotiorum* but returned to base levels after two to three days (Chen et al. 2020). Indolic glucosinolates increased in Chinese cabbage (*Brassica rapa* L. ssp. *pekinensis* cv. Kantonner) five days after infection by *A. brassicae* (Rostás et al. 2002). Thus, the time-course is obviously critical for induction responses of these defense metabolites.

Thickening of cell walls is very important for plant defense at an early stage of fungal infections (Morkunas and Ratajczak 2014). Peroxidases have been suggested to play a pivotal role in the formation of barriers against pathogens, as they lead to a polymerization of cell wall components. Moreover, they are involved in the hypersensitive reaction of cells and protect against damage caused by oxidative species. The activity of antioxidant enzymes such as peroxidase has been shown to increase with increasing fertilization (Dietrich et al. 2004; Yue et al. 2021). In contrast, fertilization did not influence the peroxidase activity of *B. orientalis* plants. However, in response to *A. brassicae*, peroxidase activity increased significantly in our experiment. Likewise, an induction of peroxidase activity in response to *A. brassicae* has been found in several other Brassicaceae species (Mallick et al. 2015; Rostás et al. 2002). The regulation of peroxidase in response to fungal infection is closely linked with sugar signaling (Morkunas and Ratajczak 2014). In response to fungal infection, we found a moderate positive relationship between fructose content and peroxidase activity in *B. orientalis*. This suggests a role of fructose as a signal molecule in regulating plant defense mechanisms (Formela et al. 2014).

Leaves of *B. orientalis* are also characterized by a relatively high density of trichomes compared to other Brassicaceae (Travers-Martin and Müller 2008), but densities largely vary between populations of this species (Fortuna et al. 2014). Trichomes can act as effective mechanical defense against many plant antagonists (Elad and Evensen 1995; Tian et al. 2012). The trichome density on both leaf sides was significantly enhanced in highly fertilized *B. orientalis* plants in the present study, suggesting that the investment in trichomes may be energy-demanding. Nitrogen fertilization has been shown to enhance leaf expansion of *B. orientalis* in the field (Tewes and Müller 2018). An efficient allocation of resources to leaf development may enable the plants to also allocate some resources into the trichomes on the surface, which represent the first line of defense against environmental challenges. Higher densities of trichomes in response to high N fertilization have also been reported in other species (Bilkova et al. 2016; Zettlemoyer 2022).

In the plant-herbivore experiment 1, aboveground biomass was likewise enhanced by fertilization, as expected. However, as in the plant-pathogen experiment, fertilization had no influence on N content, C/N ratio and total glucosinolate concentration in *B. orientalis*. Likewise, the herbivore treatment did not affect any of these plant traits. The herbivore damage for two days may have been too short and too little damage may have been imposed in the confined area of the clip cage to cause any significant changes. Likewise, a five day feeding by 6 larvae (first instar) of *M. brassicae* did not lead to an induction of leaf glucosinolates in two other populations of *B. orientalis* (Harvey and Gols 2011). In contrast, when 10 larvae of *M. brassicae* fed for a longer time (four weeks) on *B. orientalis* leaves, a significant increase in glucosinolates, particularly of *p*-hydroxybenzyl glucosinolate and 1-methylethyl glucosinolate, was found (Fortuna et al. 2014). Thus, at least under short herbivore exposure, *B. orientalis* may rather rely on mechanical defense, e.g. by high trichome density, but may induce chemical defenses only after long-term exposure. Other defense chemicals, not investigated here, may have been induced more rapidly.

The plant nutritional quality is critical for the growth and survival of herbivores, affecting their metabolism (Lu et al. 2007). Conversely to our expectation, host plant growth conditions under different supply of N fertilization did not affect the larval performance and survival of *M. brassicae*. The larval biomass gain increased with time, indicating that *B. orientalis* is in general a suitable host for larvae of *M. brassicae*. This herbivore has also been found in the field on *B. orientalis* and larvae develop well on this plant species (Harvey et al. 2010). However, under natural conditions, young larvae of *M. brassicae* usually feed gregariously (CABI 2022), thus our design of placing just one larva on a plant was somewhat artificial. An initial feeding of more young larvae may have caused stronger effects on older larvae, with different effects in dependence of nitrate fertilization. As a generalist herbivore with a very broad host range, *M. brassicae* may be less sensitive towards different fertilization treatments and be able to maintain growth and development simultaneously. The larval performance and survival of herbivores can vary greatly under different fertilization treatments on different plant species (Casey and Raupp 1999; Lu et al. 2007; Salehi et al. 2020).

Since enhanced fertilization mostly affected aboveground biomass and trichome density of the leaves rather than induction of chemical defenses in response to antagonist attack, it may be concluded that plants of *B. orientalis* tested here invest rather into biomass and morphological defense than chemical defense. Future studies may investigate, how *B. orientalis* respond to a simultaneous or more pronounced attack of different antagonists and whether plant-mediated cross-effects occur between *A. brassicae* and *M. brassicae*. In response to the low levels of fungal infection and herbivore attack as tested here the plants of this invasive population may be quite robust and able to coordinate their resource allocation well.

## Electronic Supplementary Material

Below is the link to the electronic supplementary material.

Supplementary Material 1

Supplementary Material 2

## Data Availability

Raw data are presented in the Supplement Table S3 (pathogen data) and S4 (herbivore data).

## References

[CR1] Agerbirk N, Olsen CE (2012). Glucosinolate structures in evolution. Phytochemistry.

[CR2] Anjum NA, Gill SS, Umar S, Ahmad I, Duarte AC, Pereira E (2012). Improving growth and productivity of oleiferous Brassicas under changing environment: significance of nitrogen and sulphur nutrition, and underlying mechanisms. Sci World J.

[CR3] Bates D, Mächler M, Bolker B, Walker S (2015). Fitting linear mixed-effects models using lme4. J Stat Softw.

[CR4] Berger S, Sinha AK, Roitsch T (2007). Plant physiology meets phytopathology: plant primary metabolism and plant–pathogen interactions. J Exp Bot.

[CR5] Bilkova I, Kjaer A, van der Kooy F, Lommen WJM (2016) Effects of N fertilization on trichome density, leaf size and artemisinin production in *Artemisia annua* leaves. Acta Hortic 369–376. doi:10.17660/ActaHortic.2016.1125.48

[CR6] Birnbaum C (2006) Invasive alien species fact sheet *Bunias orientalis*. – From: Online Database of the European Network on Invasive Alien Species – NOBANIS wwwnobanisorg, Date of access 02/02/2021

[CR7] Bonfig KB, Schreiber U, Gabler A, Roitsch T, Berger S (2006). Infection with virulent and avirulent *P. syringae* strains differentially affects photosynthesis and sink metabolism in *Arabidopsis* leaves. Planta.

[CR8] Bostock RM, Karban R, Thaler JS, Weyman PD, Gilchrist D (2001). Signal interactions in induced resistance to pathogens and insect herbivores. Eur J Plant Pathol.

[CR9] Brooks ME, Kristensen K, van Benthem KJ, Magnusson A (2017). GlmmTMB balances speed and flexibility among packages for zero-inflated generalized linear mixed modeling. R J.

[CR10] Bryant JP, Chapin FS, Klein DR (1983). Carbon/nutrient balance of boreal plants in relation to vertebrate herbivory. Oikos.

[CR11] CABI (2022). Mamestra brassicae. invasive species compendium.

[CR12] Casey CA, Raupp MJ (1999). Supplemental nitrogen fertilization of containerized azalea does not affect performance of Azalea Lace bug (Heteroptera: Tingidae). Environ Entomol.

[CR13] Chen J, Ullah C, Giddings Vassão D, Reichelt M, Gershenzon J, Hammerbacher A (2020). *Sclerotinia sclerotiorum* infection triggers changes in primary and secondary metabolism in *Arabidopsis thaliana*. Phytopathology.

[CR14] Chen X-j, Zhu Z-j, Ni X-l, Qian Q-q (2006) Effect of nitrogen and sulfur supply on glucosinolates in *Brassica campestris* ssp. *chinensis*. Agric Sci China 5:603–608. doi:10.1016/S1671-2927(06)60099-0

[CR15] Chen Y, Olson DM, Ruberson JR (2010). Effects of nitrogen fertilization on tritrophic interactions. Arthropod-Plant Interact.

[CR16] Cho Y (2015). How the necrotrophic fungus *Alternaria brassicicola* kills plant cells remains an enigma. Eukaryot Cell.

[CR17] Cipollini ML, Paulk E, Cipollini DF (2002). Effect of nitrogen and water treatment on leaf chemistry in horsenettle (*Solanum carolinense*), and relationship to herbivory by flea beetles (*Epitrix spp*) and tobacco hornworm (*Manduca sexta*). J Chem Ecol.

[CR18] Cooper JB, Varner JE (1984). Cross-linking of soluble extensin in isolated cell walls. Plant Physiol.

[CR19] Dietrich R, Ploß K, Heil M (2004). Constitutive and induced resistance to pathogens in *Arabidopsis thaliana* depends on nitrogen supply. Plant Cell Environ.

[CR20] Ding S, Shao X, Li J, Ahammed GJ (2021). Nitrogen forms and metabolism affect plant defence to foliar and root pathogens in tomato. Plant Cell Environ.

[CR21] Elad Y, Evensen K (1995). Physiological aspects of resistance to *Botrytis cinerea*. Phytopathology.

[CR22] Esmeijer-Liu AJ, Aerts R, Kürschner WM, Bobbink R, Lotter AF, Verhoeven JTA (2009). Nitrogen enrichment lowers *Betula pendula* green and yellow leaf stoichiometry irrespective of effects of elevated carbon dioxide. Plant Soil.

[CR23] Farr, D.F., and Rossman, A.Y (2022) Fungal databases, U.S. National Fungus Collections, ARS, USDA. Retrieved Accessed August 26, 2022, from https://nt.ars-grin.gov/fungaldatabases/

[CR24] Fischer K, Fiedler K (2000). Response of the copper butterfly *Lycaena tityrus* to increased leaf nitrogen in natural food plants: evidence against the nitrogen limitation hypothesis. Oecologia.

[CR25] Formela M, Samardakiewicz S, Marczak Ł, Nowak W (2014). Effects of endogenous signals and *Fusarium oxysporum* on the mechanism regulating genistein synthesis and accumulation in yellow lupine and their impact on plant cell cytoskeleton. Molecules.

[CR26] Fortuna TM, Eckert S, Harvey JA, Vet LE, Müller C, Gols R (2014). Variation in plant defences among populations of a range-expanding plant: consequences for trophic interactions. New Phytol.

[CR27] Fox J, Weisberg S (2019) An R Companion to applied regression. Third edition. Sage, Thousand Oaks CA

[CR28] Glazebrook J, Roby D (2018). Plant biotic interactions: from conflict to collaboration. Plant J.

[CR29] Gorden NLS, Adler LS (2013). Abiotic conditions affect floral antagonists and mutualists of *Impatiens capensis* (Balsaminaceae). Am J Bot.

[CR30] Grimmer MK, John Foulkes M, Paveley ND (2012). Foliar pathogenesis and plant water relations: a review. J Exp Bot.

[CR31] Guo J, Jia Y, Chen H, Zhang L (2019). Growth, photosynthesis, and nutrient uptake in wheat are affected by differences in nitrogen levels and forms and potassium supply. Sci Rep.

[CR32] Halkier BA, Gershenzon J (2006). Biology and biochemistry of glucosinolates. Annu Rev Plant Biol.

[CR33] Hamilton JG, Zangerl AR, DeLucia EH, Berenbaum MR (2001). The carbon-nutrient balance hypothesis: its rise and fall. Ecol Lett.

[CR34] Hartig F (2020) DHARMa: Residual Diagnostics for Hierarchical (Multi-Level / Mixed) Regression Models. Version 0.3.3.0. http://florianhartig.github.io/DHARMa/, Date of access 02.09.2020

[CR35] Harvey JA, Biere A, Fortuna T, Vet LEM (2010). Ecological fits, mis-fits and lotteries involving insect herbivores on the invasive plant, *Bunias orientalis*. Biol Invasions.

[CR36] Harvey JA, Gols R (2011). Development of *Mamestra brassicae* and its solitary endoparasitoid *Microplitis mediator* on two populations of the invasive weed *Bunias orientalis*. Popul Ecol.

[CR37] He W-M, Yu G-L, Sun Z-K (2011). Nitrogen deposition enhances *Bromus tectorum* invasion: biogeographic differences in growth and competitive ability between China and North America. Ecography.

[CR38] Herbers K, Takahata Y, Melzer M, Mock HP, Hajirezaei M, Sonnewald U (2000). Regulation of carbohydrate partitioning during the interaction of potato virus Y with tobacco. Molec Plant Pathol.

[CR39] Herms DA, Mattson WJ (1992). The Dilemma of plants: to grow or defend. Q Rev Biol.

[CR40] Hoagland DR, Arnon DI (1950). The water-culture method for growing plants without soil. Circ -Calif Agric Exp Stn.

[CR41] Hsu Y-T, Shen T-C, Hwang S-Y (2009). Soil fertility management and pest responses: a comparison of organic and synthetic fertilization. J Econ Entomol.

[CR42] Kessler A, Baldwin IT (2002). Plant responses to insect herbivory: the emerging molecular analysis. Annu Rev Plant Biol.

[CR43] Kováts E (1958). Gas-chromatographische Charakterisierung organischer Verbindungen. Teil 1: Retentionsindices aliphatischer Halogenide, Alkohole, Aldehyde und Ketone. Helv Chim Acta.

[CR44] Kurze S, Heinken T, Fartmann T (2018). Nitrogen enrichment in host plants increases the mortality of common Lepidoptera species. Oecologia.

[CR45] Kutyniok M, Müller C (2013). Plant-mediated interactions between shoot-feeding aphids and root-feeding nematodes depend on nitrate fertilization. Oecologia.

[CR46] Lecompte F, Abro MA, Nicot PC (2013). Can plant sugars mediate the effect of nitrogen fertilization on lettuce susceptibility to two necrotrophic pathogens: *Botrytis cinerea* and *Sclerotinia sclerotiorum*. Plant Soil.

[CR47] Leishman MR, Thomson VP (2005). Experimental evidence for the effects of additional water, nutrients and physical disturbance on invasive plants in low fertility Hawkesbury Sandstone soils, Sydney, Australia. J Ecol.

[CR48] Li S, Schonhof I, Krumbein A, Li L, Stützel H, Schreiner M (2007). Glucosinolate concentration in turnip (*Brassica rapa* ssp. *rapifera* L.) roots as affected by nitrogen and sulfur supply. J Agric Food Chem.

[CR49] Liu Y-H, Song Y-H, Ruan Y-L (2022). Sugar conundrum in plant–pathogen interactions: roles of invertase and sugar transporters depend on pathosystems. J Exp Bot.

[CR50] Long DH, Lee FN, TeBeest DO (2000). Effect of nitrogen fertilization on disease progress of rice blast on susceptible and resistant cultivars. Plant Dis.

[CR51] Lou Y, Baldwin IT (2004). Nitrogen supply influences herbivore-induced direct and indirect defenses and transcriptional responses in *Nicotiana attenuata*. Plant Physiol.

[CR52] Lü X-T, Kong D-L, Pan Q-M, Simmons ME, Han X-G (2012). Nitrogen and water availability interact to affect leaf stoichiometry in a semi-arid grassland. Oecologia.

[CR53] Lu Z-x, Yu X-p, Heong K-l, Hu C (2007). Effect of nitrogen fertilizer on herbivores and its stimulation to major insect pests in rice. Rice Sci.

[CR54] Mallick SA, Kumari P, Gupta M, Gupta S (2015). Effect of *Alternaria* blight infection on biochemical parameters, quantity and quality of oil of mustard genotypes. Ind J Plant Physiol.

[CR55] Monson RK, Trowbridge AM, Lindroth RL, Lerdau MT (2022). Coordinated resource allocation to plant growth–defense tradeoffs. New Phytol.

[CR56] Morgan JB, Connolly EL (2013). Plant-soil interactions: nutrient uptake. Nat Ed.

[CR57] Morkunas I, Ratajczak L (2014). The role of sugar signaling in plant defense responses against fungal pathogens. Acta Phys Plant.

[CR58] Mur LAJ, Simpson C, Kumari A, Gupta AK, Gupta KJ (2017). Moving nitrogen to the centre of plant defence against pathogens. Ann Bot.

[CR59] Novotny AM, Schade JD, Hobbie SE, Kay AD, Kyle M, Reich PB, Elser JJ (2007). Stoichiometric response of nitrogen-fixing and non-fixing dicots to manipulations of CO2, nitrogen, and diversity. Oecologia.

[CR60] Ohyama T (2010). Nitrogen as a major essential element of plants. Nitrogen Assimilation in Plants Ohyama T SK.

[CR61] Pedras MSC, Montaut S, Zaharia IL, Gai Y, Ward DE (2003). Transformation of the host-selective toxin destruxin B by wild crucifers: probing a detoxification pathway. Phytochemistry.

[CR62] R Core Team (2020) R: a language and environment for statistical computing. The R Foundation for Statistical Computing, Vienna, Austria. http://www.R-project.org

[CR63] Rejeb IB, Pastor V, Mauch-Mani B (2014). Plant responses to simultaneous biotic and abiotic stress: molecular mechanisms. Plants.

[CR64] Rosa GP, Barreto MDC, Pinto D, Seca AML (2020) A green and simple protocol for extraction and application of a peroxidase-rich enzymatic extract. Methods Protoc 3. doi:10.3390/mps3020025 10.3390/mps3020025 PMC7359449 32224955

[CR65] Rosa M, Prado C, Podazza G, Interdonato R, González JA, Hilal M, Prado FE (2009). Soluble sugars-metabolism, sensing and abiotic stress: a complex network in the life of plants. Plant Signal Behav.

[CR66] Rostás M, Bennett R, Hilke M (2002). Comparative physiological responses in Chinese cabbage induced by herbivory and fungal infection. J Chem Ecol.

[CR67] Salehi F, Gharekhani G, Shirazi J, Vaez N (2020). Effect of host plant cultivar and nitrogen fertilization on life history of *Helicoverpa armigera* (Lepidoptera: Noctuidae). J Plant Protect Res.

[CR68] Scharte J, SCHÖN H, Weis E (2005). Photosynthesis and carbohydrate metabolism in tobacco leaves during an incompatible interaction with *Phytophthora nicotianae*. Plant Cell Environ.

[CR69] Simon J, Gleadow RM, Woodrow IE (2010). Allocation of nitrogen to chemical defence and plant functional traits is constrained by soil N. Tree Physiol.

[CR70] Söchting HP, Verreet JA (2004). Effects of different cultivation systems (soil management, nitrogen fertilization) on the epidemics of fungal diseases in oilseed rape (*Brassica napus* L. var. *napus*). J Plant Dis Prot.

[CR71] Solomon PS, Tan K-C, Oliver RP (2003). The nutrient supply of pathogenic fungi; a fertile field for study. Mol Plant Pathol.

[CR72] Steinlein T, Dietz H, Ullmann I (1996). Growth patterns of the alien perennial *Bunias orientalis* L (Brassicaceae) underlying its rising dominance in some native plant assemblages. Vegetatio.

[CR73] Sun Y, Wang M, Mur LAJ, Shen Q, Guo S (2020) Unravelling the roles of nitrogen nutrition in plant disease defences. Int J Mol Sci 21. doi:10.3390/ijms21020572 10.3390/ijms21020572 PMC7014335 31963138

[CR74] Tewes LJ, Michling F, Koch MA, Müller C (2018). Intracontinental plant invader shows matching genetic and chemical profiles and might benefit from high defence variation within populations. J Ecol.

[CR75] Tewes LJ, Müller C (2018). Syndromes in suites of correlated traits suggest multiple mechanisms facilitating invasion in a plant range-expander. Neobiota.

[CR76] Tewes LJ, Müller C (2020). Interactions of *Bunias orientalis* plant chemotypes and fungal pathogens with different host specificity in vivo and in vitro. Sci Rep.

[CR77] Therneau T(2022) A package for survival analysis in R. R Package Version 3.3-1. https://cran.r-project.org/web/packages/survival/index.html, Date of access 03/03/2022.

[CR78] Tian D, Tooker J, Peiffer M, Chung SH, Felton GW (2012). Role of trichomes in defense against herbivores: comparison of herbivore response to woolly and hairless trichome mutants in tomato (*Solanum lycopersicum*). Planta.

[CR79] Travers-Martin N, Müller C (2008). Specificity of induction responses in *Sinapis alba* L. Plant Signal Behav.

[CR80] van Dijk LJA, Ehrlén J, Tack AJM (2021). Direct and insect-mediated effects of pathogens on plant growth and fitness. J Ecol.

[CR81] Vega A, Canessa P, Hoppe G, Retamal I (2015). Transcriptome analysis reveals regulatory networks underlying differential susceptibility to *Botrytis cinerea* in response to nitrogen availability in *Solanum lycopersicum*. Front Plant Sci.

[CR82] Wang H, Hutwimmer S, Strasser H, Burgstaller W (2009). Destruxin production of *Metarhizium anisopliae* under carbon and nitrogen exhaustion. J Basic Microbiol.

[CR83] Wei J, Yan L, Ren QIN, Li C, Ge F, Kang LE (2013). Antagonism between herbivore-induced plant volatiles and trichomes affects tritrophic interactions. Plant Cell Environ.

[CR84] Yue K, Li L, Xie J, Fudjoe SK, Zhang R, Luo Z, Anwar S(2021) Nitrogen supply affects grain yield by regulating antioxidant enzyme activity and photosynthetic capacity of maize plant in the Loess Plateau. Agronomy 11. doi:10.3390/agronomy11061094

[CR85] Zettlemoyer MA (2022). Leaf traits mediate herbivory across a nitrogen gradient differently in extirpated vs. extant prairie species. Oecologia.

