# Supplementary material for: Responses of Bunias orientalis to Short-term Fungal Infection and Insect Herbivory are Independent of Nutrient Supply
Source: J Chem Ecol. 2022 Nov 19;48(11-12):827–40. doi: 10.1007/s10886-022-01392-0 (PMC9840571; doi:10.1007/s10886-022-01392-0)
Supplement: Supplementary file 1 — Supplementary Material 1 [file 10886_2022_1392_MOESM1_ESM.pdf]

**Supplement to:**

# Responses of *Bunias orientalis* to Short-term Fungal Infection and Insect Herbivory are Independent of Nutrient Supply

Blaise Binama[1], Miriam Behrendt[1], Caroline Müller[1]*

[1] Department of Chemical Ecology, Bielefeld University, Universitätsstr. 25, 33615 Bielefeld, Germany

* Corresponding author. E-mail: caroline.mueller@uni-bielefeld.de

**Supplement Table S1**: Mineral nutrient solution used to fertilize *Bunias orientalis* plants with either low [1 mM $Ca(NO_3)_2$] or high [4 mM $Ca(NO_3)_2$] nitrate treatment.

| **Macronutrients** | final concentration |
|---|---|
| $Ca(NO_3)_2$ | 1 mM (low) or 4 mM (high) |
| KCl | 1 mM |
| $KH_2PO_4$ | 0.5 mM |
| $MgSO_4$ | 1 mM |
| Fe(III)-citrate | 0.05 mM |
| **Micronutrients** | |
| $Na_2MoO_4$ | 0.2 µM |
| $H_3BO_3$ | 10 µM |
| $NiCl_2$ | 0.2 µM |
| $ZnSO_4$ | 1 µM |
| $MnCl_2$ | 0.2 µM |
| $CuSO_4$ | 0.5 µM |
| $CoCl_2$ | 0.2 µM |

**Supplement Table S2**: Effects of fertilization and pathogen infection on contents of different soluble sugars in treated leaves of *Bunias orientalis*.

| | Fertilization | | | Infection | | | Fertilization * Infection | | |
|---|---|---|---|---|---|---|---|---|---|
| | *df* | $\chi 2$ | *P* | *df* | $\chi 2$ | *P* | *df* | $\chi 2$ | *P* |
| Total sugars | 1 | 0.13 | 0.717 | 1 | 10.99 | 0.001 | 1 | 0.00 | 0.997 |
| Glucose (sqrt) | 1 | 0.51 | 0.474 | 1 | 2.59 | 0.107 | 1 | 0.29 | 0.588 |
| Fructose (sqrt) | 1 | 0.43 | 0.51 | 1 | 11.86 | 0.001 | 1 | 0.01 | 0.936 |
| Sucrose (sqrt) | 1 | 1.72 | 0.19 | 1 | 3.63 | 0.057 | 1 | 0.77 | 0.379 |

Traits were analyzed using linear mixed-effects models. All models were fitted using the maximum likelihood method, and *P* values were determined using likelihood ratio tests (chi-square tests); *df* = degrees of freedom. Fertilization (low or high) and Infection (control or fungus).

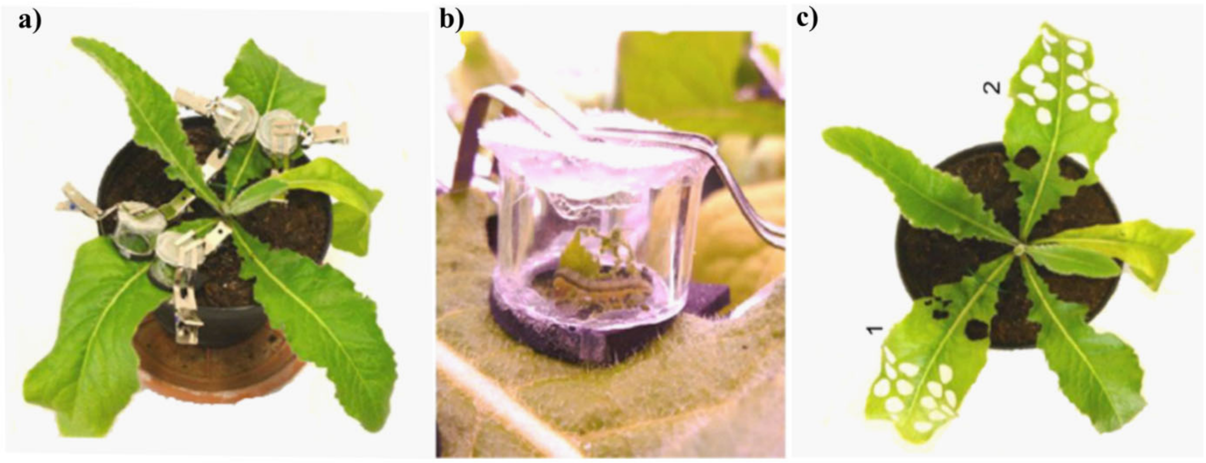


**Figure S1**: Set-up of plant-herbivore experiment 1 using *Mamestra brassicae* larvae on plants of *Bunias orientalis* to test for plant responses. (a) clip cages attached to the leaf base of the youngest, fully developed leaf pair, (b) 15-day-old larva placed in a clip cage for 48 h, after which harvest took place immediately and (c) harvest scheme for chemical analysis. Numbers indicate the areas from which leaf discs were removed for analysis of (1) glucosinolates and C/N (determined from control plants only) or (2) harvested as a back-up.

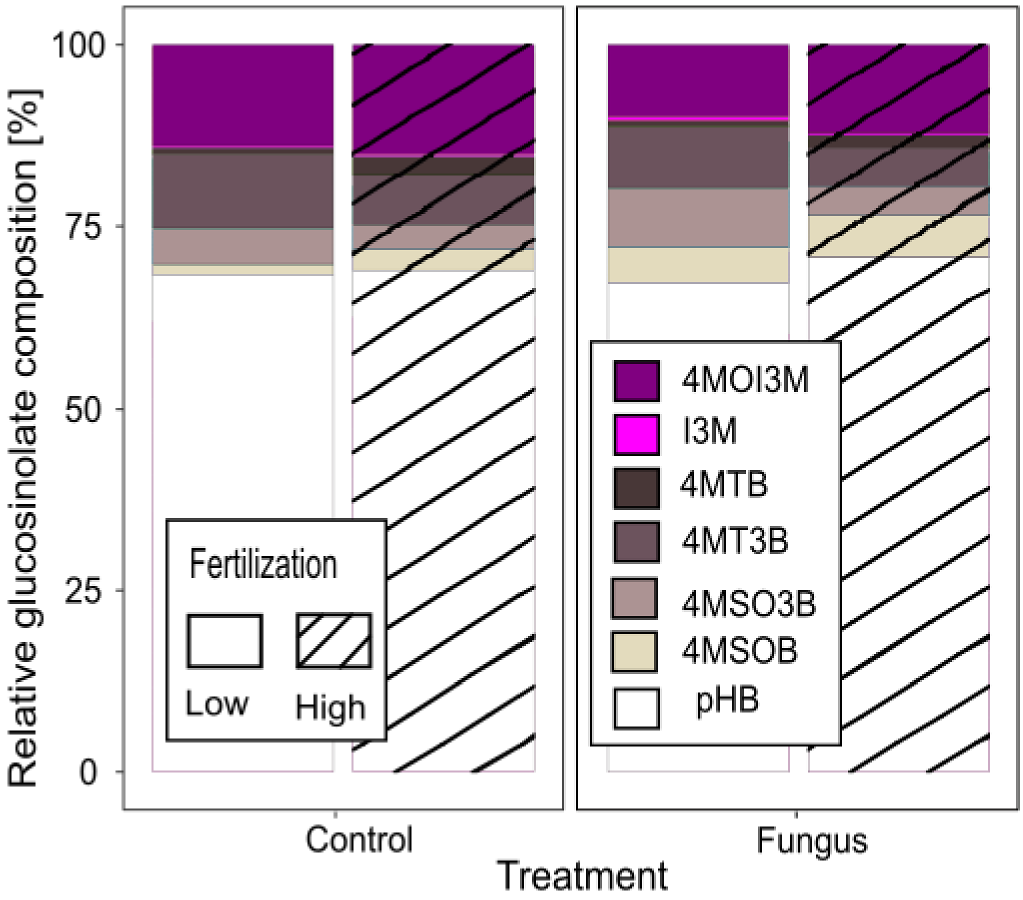


**Figure S2**: Relative glucosinolate (GS) composition of leaves of *Bunias orientalis* kept uninfected (control) or infected with the fungus *Alternaria brassicae* in plants grown under low or high fertilization, including one benzenic GS (white bar unit; pHB, *p*-hydroxybenzyl GS), aliphatic GSs (grey bar units; 4MSO3B, 4-methylsulfinyl-3-butyl GS; 4MSOB, 4-methylsulfinylbutyl GS; 4MTB, 4-methylthiobutyl GS; 4MT3B, 4-methylthio-3-butenyl GS), and indole GSs (purple bar units; I3M, indol-3-ylmethyl GS; 4MOI3M, 4-methoxyindol-3-ylmethyl GS). Values are averaged across 14-15 replicates.

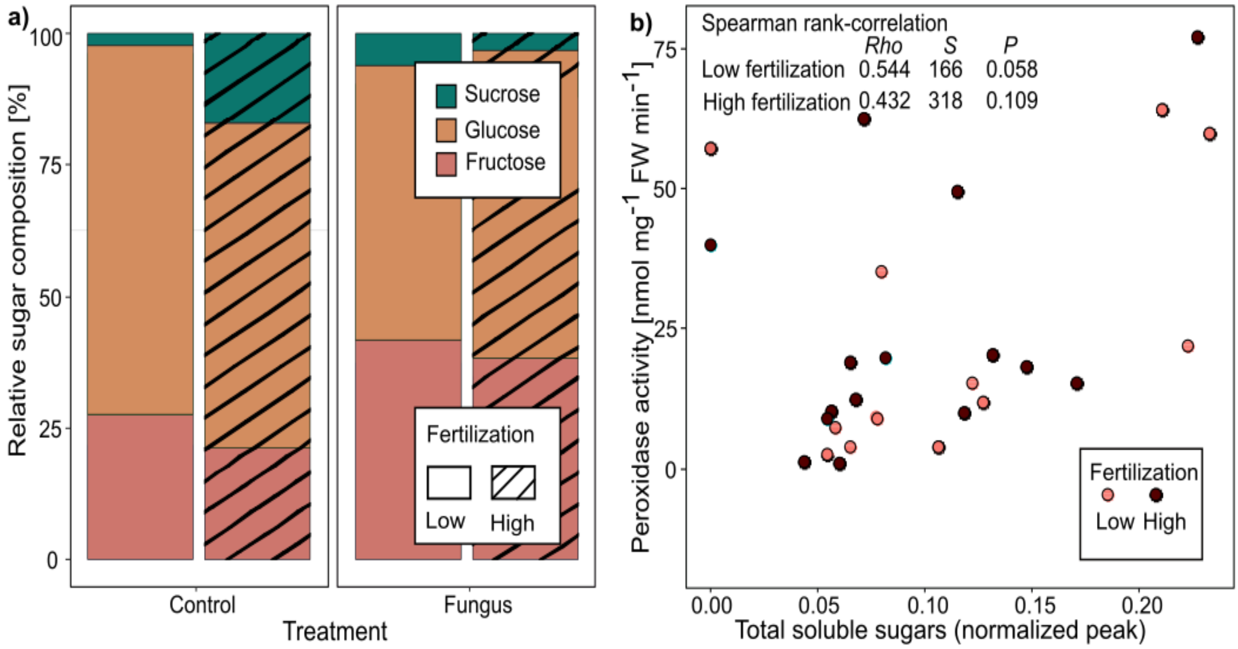


**Figure S3:** (a) Relative soluble sugar composition of leaves kept uninfected (control) or infected with the fungus *Alternaria brassiceae* (infection site included) of plants grown under low or high fertilization, including glucose, fructose and sucrose (averaged across 13-15 replicates); (b) correlation between fructose content (including infection site) and peroxidase enzyme activity (distal from infection site) in leaves of *Bunias orientalis* infected with the fungus *Alternaria brassicae* of plants grown under low and high fertilization; rho: coefficient of determination (Spearman rank correlation).

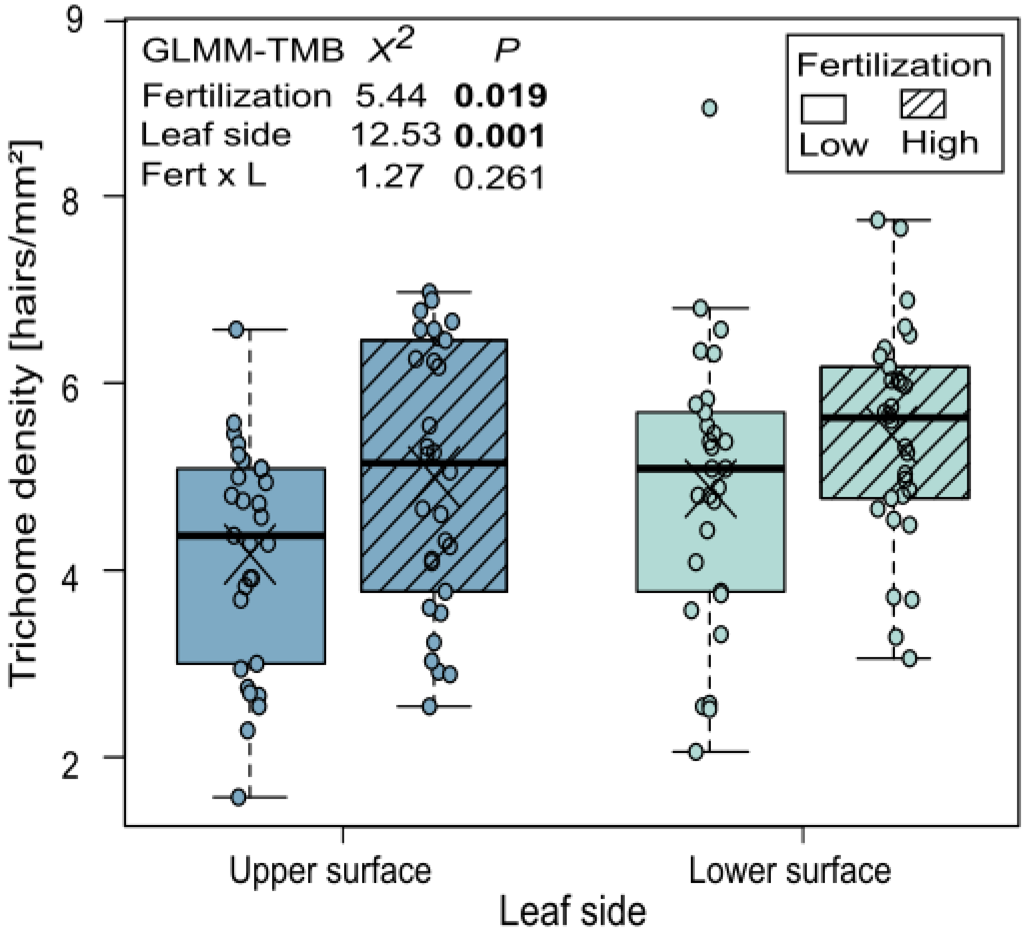


**Figure S4**: (a) Trichome density (number per mm$^2$) of *Bunias orientalis* leaves of plants kept under low and high fertilization. The statistical analysis of the effects of fertilization (Fert) and leaf side (L) is based on raw data (number per 56.6 mm$^2$) and was analyzed using a generalized linear mixed model (GLMM), fitted with glmm Template Model Builder; n = 29-30.

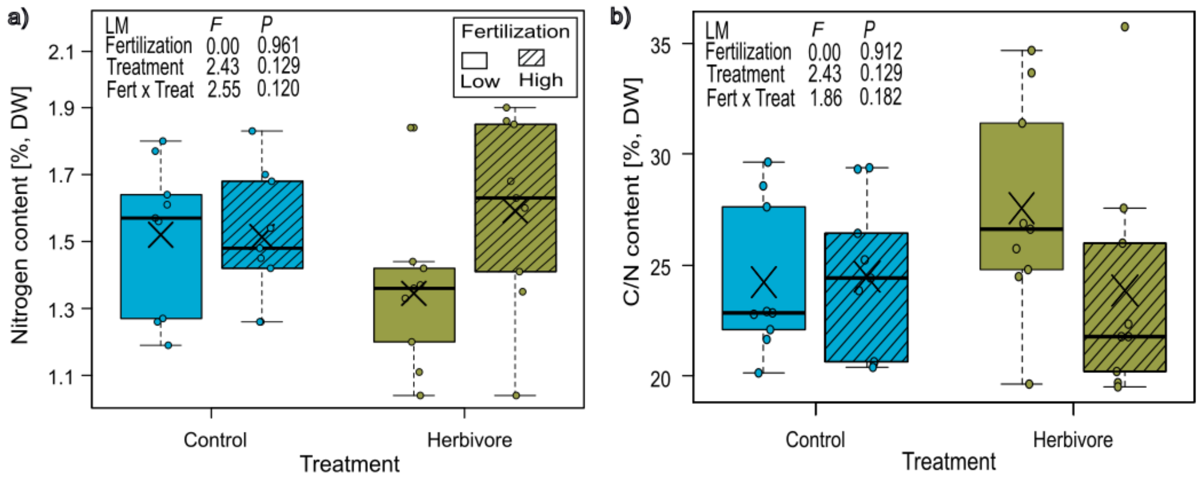


**Figure S5**: Responses of plants of *Bunias orientalis* to nitrate fertilization (Fert, low or high) in (a) nitrogen content and (b) carbon to nitrogen ratio of uninfested leaves (control) or infested (Treatment – Treat) with larvae of *Mamestra brassicae* (herbivore; plants of plant-herbivore experiment 1). Traits were analyzed using linear models, n = 9.

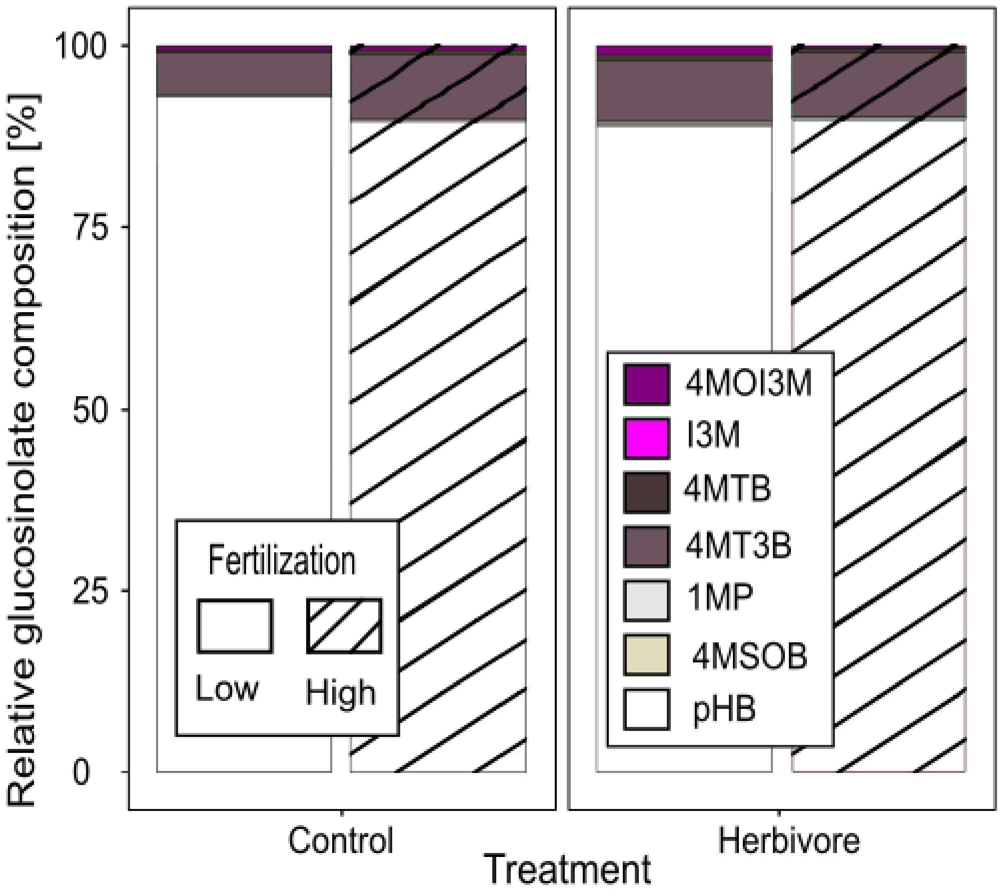


**Figure S6**: Relative glucosinolate composition of leaves of *Bunias orientalis* kept unifested (control) or infested with larvae of *Mamestra brassicae* in plants grown under low or high fertilization, including one benzenic GS (white bar unit; pHB, *p*-hydroxybenzyl GS), aliphatic GSs (grey bar units; 1-methylpropyl GS; 4MSOB, 4-methylsulfinylbutyl GS; 4MTB, 4-methylthiobutyl GS; 4MT3B, 4-methylthio-3-butenyl GS), and indole GSs (purple bar units; I3M, indol-3-ylmethyl GS; 4MOI3M, 4-methoxyindol-3-ylmethyl GS). Values are averaged across 9 replicates.
